# Treatment of nocturnal leg cramps by blockade of the medial branch of the deep peroneal nerve after lumbar spine surgery

**DOI:** 10.1002/brb3.370

**Published:** 2015-08-07

**Authors:** Takayuki Imura, Gen Inoue, Toshiyuki Nakazawa, Masayuki Miyagi, Wataru Saito, Kentaro Uchida, Takanori Namba, Eiki Shirasawa, Naonobu Takahira, Masashi Takaso

**Affiliations:** 1 Department of Orthopaedic Surgery, School of Medicine, Kitasato UniversitySagamihara, Japan; 2 Department of Rehabilitation, School of Allied Health Sciences, Kitasato UniversitySagamihara, Japan

**Keywords:** Deep peroneal nerve, leg cramp, lumbar spine surgery, peripheral nerve blockade

## Abstract

**Introduction:**

Patients with lumbar spine disease sometimes complain of nocturnal leg cramps. We sought to investigate the effectiveness of blocking the medial branch of the deep peroneal nerve as treatment for nocturnal leg cramps after spinal surgery for lumbar spine disease.

**Methods:**

We evaluated 66 postoperative patients in this prospective comparative study of a group of patients with a nerve block (*n* = 41) and a control group without (*n* = 25). In the block group, the medial branch of the deep peroneal nerve was blocked at the distal two-thirds of the interspace between the first and second metatarsals using 5.0 mL of 1.0% lidocaine.

**Results:**

Two weeks after the block, the frequency of nocturnal leg cramps was reduced to less than a quarter of pretreatment baseline frequency in 61.0% of patients (*n* = 25) and less than half in 80.5% (*n* = 33). In the control group, the frequency of the leg cramps was reduced from baseline in 32.0% of patients (*n* = 8), and was unchanged or increased in 68.0% (*n* = 17) at 2 weeks. Cramp frequency was reduced to less than a quarter or less than half of baseline frequency in a significantly (*P *<* *0.05 and *P *<* *0.01, respectively) larger percentage of patients in the block group. The severity of each cramp was less in about two-thirds of patients (63.4%; *n* = 26) in the block group and was unchanged in one-third (31.7%; *n* = 13).

**Conclusions:**

Blocking the medial branch of the peroneal nerve can be an effective, long-lasting, and simple treatment with low risk for nocturnal cramps sustained after lumbar spine surgery.

## Introduction

Patients with lumbar spine disease sometimes complain of nocturnal leg cramps, defined as acute, variable, and involuntary painful contractions of the muscles in the lower extremities. Leg cramps may affect populations ranging from the young athlete to the elderly (Young et al. 1989; Naylor and Young 1994; Dahle et al. 1995; Abdulla et al. 1999; Butler et al. 2002; Miller and Layzer 2005; Robertson et al. 2005; Shaker et al. 2005; Vinciguerra et al. 2006). Leg cramps are associated with neuromuscular diseases, diabetes, hyperthyroidism, hypertension, hypocalcaemia, hyperkalemia, vascular diseases, and some medications including diuretics, statins, and steroids (Abdulla et al. 1999; Kanaan and Sawaya 2001; Vinciguerra et al. 2006). Leg cramps are strongly related to lumbar spinal stenosis (LSS) and surgical interventions do not improve leg cramps in patients with LSS (Matsumoto et al. 2009). Consequently, leg cramps may be a chronic complaint of such patients, and continue to disturb their quality of life, even if the surgery was successful in treating their lumbar spine disease.

Treatment of leg cramps has included various methods in an attempt to improve sleep. Physiological treatment has involved stretch exercises and foot splints worn at night (Man-Son-Hing et al. 1998; Blyton et al. 2012; Hallegraeff et al. 2012). Pharmacological treatments have included quinine, magnesium citrate, vitamin D3, tizanidine hydrochloride, eperisone hydrochloride, vitamin B, vitamin C, vitamin E, taurine, epalrestat, gabapentin, verapamil, carisoprodol, orphenadrine, and Shakuyaku-kanzo-to (a Chinese herbal medicine) (Fowler 1973; Daniel 1979; Pitkin 1983; Warburton et al. 1987; Sontag and Wanner 1988; Yamamoto 1994; Chan et al. 1998; Abdulla et al. 1999; Serrao et al. 2000; Khajehdehi et al. 2001; Roffe et al. 2002; Hinoshita et al. 2003; Miller and Layzer 2005; Guay 2008; Malanga et al. 2008; Ziegler 2008; El-Tawil et al. 2010; Chandanwale et al. 2011; Chandok et al. 2011; Blyton et al. 2012; Hallegraeff et al. 2012). In Japan, peripheral nerve block has been used as a treatment for nocturnal leg cramps. Peripheral block of branches of the deep peroneal nerve is an effective treatment for leg cramps (Takayama and Ito 2002; Yuda et al. 2005; Imura et al. 2015). However, no reports have evaluated the use of peripheral nerve block as a treatment for nocturnal leg cramps following lumbar surgery. In the current study, we evaluated the effectiveness of blocking the medial branch of the deep peroneal nerve for chronic leg cramps in patients after lumbar spine surgery.

## Patients and Methods

A total of 66 patients (35 men, 31 women; mean age 60.9 years, range 28–82 years) complaining of nocturnal muscle cramps after lumbar spine surgery, who visited our hospital from April 2005 to March 2010 participated in this prospective comparative study. The study protocol was approved by our institutional review board for clinical research and treatment, and written informed consent was obtained from the patient participants after explanation of the protocol. The entry criteria into our study were: (1) patients who had undergone previous lumbar spine surgery (range 1–15 years before), and whose spinal canal or foramen was confirmed with magnetic resonance imaging to be decompressed successfully; and (2) who had at least one nocturnal cramp a week before their lumbar spine surgery, and (3) who had at least one nocturnal cramp a week for at least one month postoperatively. The exclusion criteria included the following: (1) significant hepatic, respiratory, or hematological illness or unstable cardiovascular disease; (2) current use of drugs known to be associated with cramps, and (3) inability to complete evaluations scheduled because of psychiatric or physical illness. Previous lumbar spine surgery was performed in our institute and was indicated for patients with at least one of the following symptoms: intolerable sciatica, neurological intermittent claudication, and/or bladder and bowel dysfunction that disturbed the patients’ activity of daily living. Evaluation at entry included a survey of nocturnal leg cramp features, a directed neurological examination, a complete blood cell count with a leukocyte differential count, and measurement of electrolytes, calcium, creatinine, aminotransferase, and bilirubin. Patients were divided into two groups, a block group and a control group. For the block group, a block of the medial branch of the deep peroneal nerve was performed after pharmacological or physiological treatments known to relieve muscle cramps were found to be ineffective for nocturnal leg cramps. By contrast, for the control group, the block was not indicated and only pharmacological and/or physiological treatment for the leg cramps, or no treatment was performed. Whether a block was performed was decided based on indication, the patients’ desire, and the physician’s decision after informed consent. Ultimately, 41 patients were selected to receive the block and were recruited into the block group, and the 25 patients not receiving a block were recruited into the control group. In both groups, there was no change to pharmacological treatments with or without nonsteroidal anti-inflammatory drugs or prostaglandin E1, which had not been reported to affect the leg cramps, or physiological treatments, from 3 months before starting this study or during the study period. Between the two groups, age, sex, symptomatic side effects, surgical methods, and duration after lumbar spine surgery were not significantly different at baseline (Table 1999). Cramps were defined as sudden, involuntary, and painful muscle contractions accompanied by hardening of the muscle, which were variable, but lasted no longer than 15 min.

**Table 1:** Characteristics of patients

Characteristic	Control (*n* = 25)	Block (*n* = 41)	*P*-value
Age (years)	60.7 ± 13.4	61.0 ± 15.8	N.S.
Gender			
Male	17 (68.0%)	18 (43.9%)	N.S.
Female	8 (32.0%)	23 (56.1%)	
Symptomatic side			
Right	7 (28.0%)	12 (29.3%)	N.S.
Left	9 (36.0%)	10 (24.4%)	
Bilateral	9 (36.0%)	19 (46.3%)	
Surgical methods			
Nucleotomy	7 (28.0%)	4 (9.8%)	N.S.
Decompression	13 (52.0%)	26 (63.4%)	
Decompression and fusion	5 (20.0%)	11 (26.8%)	
Duration after lumbar surgery (years)	6.1 ± 1.6	6.2 ± 2.9	N.S.

Continuous variables are presented as mean ± SD; categorical variables are presented as counts (percentage).

### Blocking method

**Figure 1:**
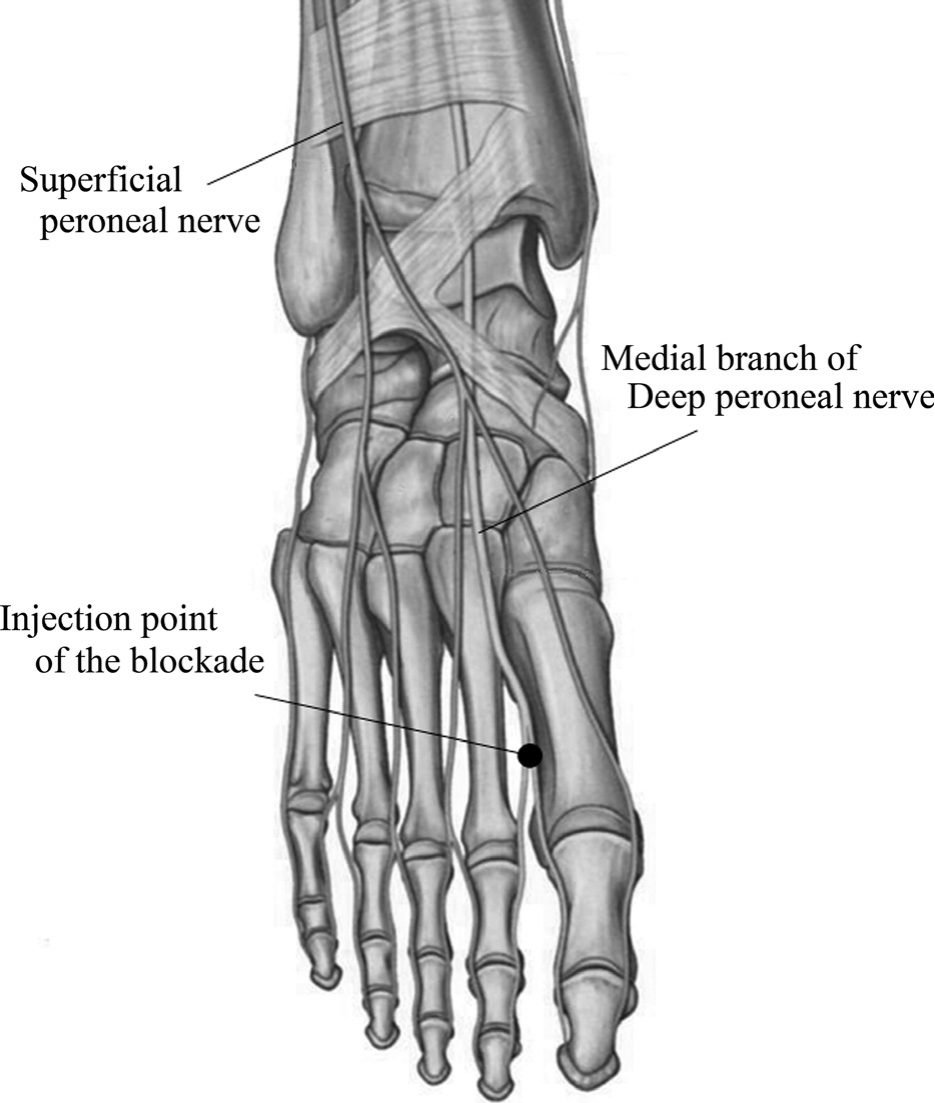
The medial terminal branch accompanies and runs medial to the dorsalis pedis artery along the dorsum of the foot. In the forefoot, it passes deep to the extensor hallucis brevis tendon, bifurcates in the midmetatarsal region, and arborizes, supplying sensibility to the first toe interspace and the adjacent sides of the first and second toes. We performed the blockade at the bifurcation of the branches, which is located in the distal two-thirds of the interspace between the first and second metatarsals.

Anatomically, the deep peroneal nerve supplies muscular branches to the tibialis anterior, extensor digitorum longus, peroneus tertius, and extensor hallucis longus, continues into the foot, along with the tibial artery and vein, and divides into lateral and medial branches at the level of the superficial fascia of the ankle. The medial terminal branch accompanies and runs medial to the dorsalis pedis artery along the dorsum of the foot. In the forefoot, it passes deep to the extensor hallucis brevis tendon, and bifurcates in the midmetatarsal region supplying sensibility to the first toe interspace and the adjacent sides of the first and second toes (Lawrence and Botte 1995; Snell 2004). We performed the nerve block at the bifurcation of the branches that is located in the distal two-thirds of the interspace between the first and second metatarsals (Fig.1). The site of injection was determined by palpation alone. The patient was placed supine with the ankle and foot in a neutral position. A 23-gauge needle was advanced slowly to a depth of from 1.0 to 1.5 cm from the skin surface. After aspiration was negative for blood, 5.0 mL of lidocaine (1% solution without epinephrine) was injected slowly over 1 or 2 min. Following the block, patients were observed for 30 min for side effects. After confirmation that no side effects had occurred, the patients were allowed to walk and were discharged. In patients with bilateral leg symptoms, bilateral medial branch blocks, each with 5.0 mL of lidocaine, were performed.

### Clinical assessment

Medical and surgical history regarding lumbar spine disease was evaluated for all patients. We determined the following three primary outcome measures that were evaluated at 2 weeks after the block, or after 2 weeks in controls without block. The frequency of nocturnal cramps was divided into five categories: (1) decreased to less than one quarter compared with baseline, (2) decreased to more than a quarter, but less than half, (3) decreased to more than half, but less than baseline, (4) was unchanged, and (5) increased. Additionally, the severity of nocturnal cramps was divided into three categories according to severity: (1) decreased compared with baseline, (2) unchanged, and (3) increased. We queried side effects if the patient experienced any problems following the block. As a secondary outcome, the effective duration of the block was determined in patients who reported reduced cramp frequency at the visit 2 weeks after the block. Patients received a medical examination once every 2 or 4 weeks for 3 months, and the time it took for the frequency to increase to the same as it was at baseline before treatment was noted.

### Statistical analysis

All calculations were performed using the statistical analysis software, JMP Pro, version 9 (SAS Institute Inc., Cary, NC). We used an unpaired Student’s *t-*test to compare age and duration after lumbar spine surgery. For categorical data, a Fisher exact test was performed to compare distribution differences between the patient groups. Differences were considered significant at *P *<* *0.05.

## Results

The changes in frequency and severity are shown in Table 1983. With a single block of the deep peroneal nerve, the frequency of nocturnal leg cramps was reduced to less than a quarter of that at baseline before treatment in 61.0% of the patients in the block group (*n* = 25) and less than half in 80.5% (*n* = 33) of the patients at 2 weeks after the block. No patients in this group reported an increase in the frequency of nocturnal leg cramps after the block. By contrast, in the control group, the frequency of nocturnal leg cramps was reported to be reduced in about one-third of patients (32.0%; *n* = 8), and unchanged in 60.0% (*n* = 15). The frequency of nocturnal leg cramps was reduced to less than a quarter or less than half of baseline frequency in a significantly (*P *<* *0.05 and *P *<* *0.01, respectively) larger percentage of patients in the block group.

**Table 2:** Changes of frequency and severity of the cramps

	Control (*n* = 25)	Block (*n* = 41)	*P*-value
Frequency			
Less than quarter	5 (20.0%)	25 (61.0%)	<0.001
Less than half but more than quarter	2 (8.0%)	8 (19.5%)	
Less but more than half	1 (4.0%)	3 (7.3%)	
No change	15 (60.0%)	5 (12.2%)	
Worse	2 (8.0%)	0 (0%)	
Severity			
Better	2 (8.0%)	26 (63.4%)	<0.001
No change	20 (80.0%)	13 (31.7%)	
Worse	3 (12.0%)	2 (4.9%)	

Categorical variables are presented as counts (percentage).

**Figure 2:**
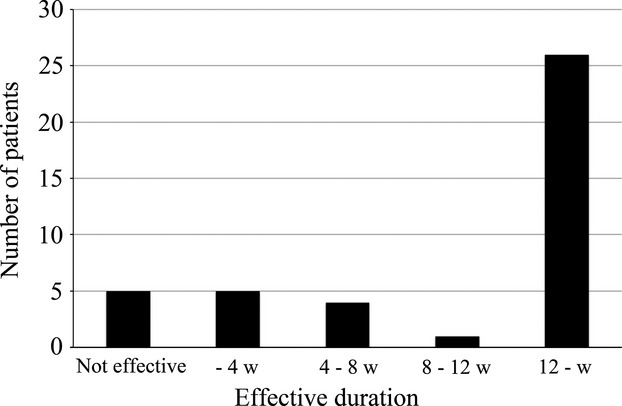
We determined the time after the block until the frequency of nocturnal leg cramps increased to the same as it was at baseline before the block. Effectiveness of the block was prolonged over 12 weeks in 63.4% of patients, but the block was completely ineffective in 12.2%, and less effective at 12 weeks in 24.4%.

The severity of each cramp was less than that before treatment in about two-thirds of patients (63.4%; *n* = 26) in the block group and unchanged in about one-third (31.7%; *n* = 13). By contrast, in the control group, the severity was unchanged in 80% of patients (*n* = 20), suggesting that the frequency and severity of the cramps are sustained for years after the surgery, and barely changes without any treatment. In 63.4% (*n* = 26) of patients in the block group, the effectiveness of the block is prolonged for over 12 weeks (Fig.2). By contrast, the block was completely ineffective in 12.2% of patients (*n* = 5) and effective for less than 12 weeks in 24.4% (*n* = 10). No adverse side effects were observed during the study or in the follow-up period in any of the 41 patients in the block group.

## Discussion

Our results show the efficacy of blocking the medial branch, which is the distal sensory nerve of the deep peroneal nerve, for treatment of chronic nocturnal leg cramps after lumbar spine surgery when pharmacological and/or physiological treatments were not effective. Nocturnal leg cramps disturb the quality of life in patients with lumbar spine disease, both preoperatively and postoperatively. Matsumoto et al. (2009) reported that leg cramps were sustained or worsened in over 70% of patients with lumbar spinal canal stenosis after decompression surgery. These authors speculated that: (1) the lumbar nerve roots were irreversibly damaged by long-lasting compression, (2) reinnervation of the affected muscles enhances the hyperexcitability of the motor units, and/or (3) most of the patients were able to walk further after surgery, leading to muscle fatigue and metabolite accumulation, which cause leg cramps. In their study, the mean postsurgical period of the patients was 6.4 years (range from 10 months to 15.5 years). This suggests that nocturnal leg cramps are a chronic problem for patients even after successful lumbar spine surgery.

The mechanism(s) of leg cramps are yet to be clarified, but disturbances in the central and peripheral nervous system and skeletal muscle could be involved (Jansen et al. 1990; Jansen et al. 1999; Miller and Layzer 2005). Electrophysiologically, cramps are characterized by repetitive firing of motor unit action potentials at rates of up to 150 per sec. This is more than four times the usual rate in maximum voluntary contraction (Bellemare et al. 1983; Jansen et al. 1990). In a human study, Ross and Thomas indicated a positive-feedback loop between peripheral afferents and alpha motor neurons, and that this loop is mediated by changes in presynaptic input. This loop is considered a possible mechanism underlying the generation of muscle cramps (Ross and Thomas 1995). The frequency of nocturnal leg cramps has also been suggested to result from changes in hydrostatic pressure and ionic shift across the cell membrane in the calf muscles in the recumbent position, inducing hyperexcitability of the motor neurons. Consequently, the pain of the cramps may be caused by an accumulation of metabolites and focal ischemia (Miller and Layzer 2005). The difference in these conditions in each patient may explain the diverse symptomatology of the cramps.

It is assumed that cramps result from spontaneous discharges of the motor nerves or abnormal excitation of the terminal branches of motor axons, which indicates the possibility of a peripheral nervous system origin (Lambert and Mulder 1957; Bertolasi et al. 1993). By contrast, Minetto et al. (2011) reported that spinal involvement is integral in cramp development and a peripheral mechanism may not be responsible. In the present study, even in patients with severe nocturnal cramps lasting for many years after lumbar spine surgery, the frequency was reduced to less than one half in about three-quarters of the patients 2 weeks after the first block, which is a significantly larger proportion compared with 28.0% of untreated control patients with a similar reduction. Moreover, the severity of cramps was less in over 60% of the patients in the block group, compared with 8.0% in the control group. Based on findings in the control group, the frequency of the cramps was less in 40% of patients and severity less in only 20%, suggesting nocturnal cramps sustained after lumbar spine surgery might only barely become less severe without any treatment within a few weeks, but that a block might affect not only the frequency but also the severity of the nocturnal cramps. The mechanism of the reduction in frequency and severity of nocturnal cramps after a block of the distal peripheral branch of the peroneal nerve remains unclear. However, a common peroneal nerve block at a point distal to the nerve injury site was effective for the pain of lumbar disc herniation (Tajiri et al. 1998; Sangwan et al. 2005). We also demonstrate that an effective site for the block is distal and distant from the site of injury. It is apparent that the local anesthetic does not reach the lesion itself, but blocks the transmission of nerve impulses by interfering with membrane depolarization (Ritchie 1975). After their double-blind study, Tajiri et al. (1998) speculated that impulses transmitted distal to the lesion are important for the generation of radicular pain, which may be treated by a nerve block. Surprisingly, the effectiveness of the block continued for more than 3 months in over 60% of the patients. Our findings could not clearly demonstrate that blockade of the distal peripheral branch with only sensory activity affects the hyperexcitability of the peripheral motor axon or a central site, such as in the spinal cord. Nevertheless, based on our findings, blocking the transmission of the nerve impulses and breaking the positive-feedback loop between peripheral afferents and alpha motor neurons, produces effective and long-lasting action to reduce both the severity and frequency of nocturnal leg cramps. However, it is possible that another unknown mechanism, unrelated to peripheral high-frequency discharges may be responsible, and is not yet elucidated or is unaffected by the block of the distal peripheral sensory branch. No patient in this study showed any adverse side effects during the study, indicating the safety of this block. However, we only evaluated the effectiveness of the block at 2 weeks. This single time-point evaluation is a limitation of the present study. Another limitation of the present study is the method used to evaluate the effectiveness of the treatment. We used only a categorical classification compared with pretreatment. The actual number of nocturnal leg cramps or a method such as a numerical rating scale (NRS) or visual analogue scale (VAS) may be more objective than our categorical classification. Further evaluation at multiple time points during a longer follow-up using various established methods is warranted. We consider that performing a deep peroneal nerve block using the medial branch approach is a new, relatively simple, and low risk therapy providing valuable treatment for patients with chronic, severe nocturnal cramps following lumbar spine surgery not treatable using other methods.

## Conflict of Interest

None declared.

## References

[b1] Abdulla AJ, Jones PW, Pearce VR (1999). Leg cramps in the elderly: prevalence, drug and disease associations. Int. J. Clin. Pract.

[b2] Bellemare F, Woods JJ, Johansson R, Bigland-Ritchie B (1983). Motor-unit discharge rates in maximal voluntary contractions of three human muscles. J. Neurophysiol.

[b3] Bertolasi L, De Grandis D, Bongiovanni LG, Zanette GP, Gasperini M (1993). The influence of muscular lengthening on cramps. Ann. Neurol.

[b4] Blyton F, Chuter V, Walter KE, Burns J (2012). Non-drug therapies for lower limb muscle cramps. Cochrane Database Syst. Rev.

[b5] Butler JV, Mulkerrin EC, O’Keeffe ST (2002). Nocturnal leg cramps in older people. Postgrad. Med. J.

[b6] Chan P, Huang TY, Chen YJ, Huang WP, Liu YC (1998). Randomized, double-blind, placebo-controlled study of the safety and efficacy of vitamin B complex in the treatment of nocturnal leg cramps in elderly patients with hypertension. J. Clin. Pharmacol.

[b7] Chandanwale AS, Chopra A, Goregaonkar A, Medhi B, Shah V, Gaikwad S (2011). Evaluation of eperisone hydrochloride in the treatment of acute musculoskeletal spasm associated with low back pain: a randomized, double-blind, placebo-controlled trial. J. Postgrad. Med.

[b8] Chandok N, Tan P, Uhanova J, Shankar N, Marotta P (2011). A pilot study of vitamin E for the treatment of cirrhotic muscle cramps. Liver Int.

[b9] Dahle LO, Berg G, Hammar M, Hurtig M, Larsson L (1995). The effect of oral magnesium substitution on pregnancy-induced leg cramps. Am. J. Obstet. Gynecol.

[b10] Daniel HW (1979). Simple cure for nocturnal leg cramps. N. Engl. J. Med.

[b11] El-Tawil S, Al Musa T, Valli H, Lunn MP, El-Tawil T, Weber M (2010). Quinine for muscle cramps. Cochrane Database Syst. Rev.

[b12] Fowler AW (1973). Relief of cramps. Lancet.

[b13] Guay DR (2008). Are there alternatives to the use of quinine to treat nocturnal leg cramps?. Consult Pharm.

[b14] Hallegraeff JM, van der Schans CP, de Ruiter R, de Greef MH (2012). Stretching before sleep reduces the frequency and severity of nocturnal leg cramps in older adults: a randomised trial. J. Physiother.

[b15] Hinoshita F, Ogura Y, Suzuki Y, Hara S, Yamada A, Tanaka N (2003). Effect of orally administered shao-yao-gan-cao-tang (Shakuyaku-kanzo-to) on muscle cramps in maintenance hemodialysis patients: a preliminary study. Am. J. Chin. Med.

[b16] Imura T, Inoue G, Nakazawa T, Ueno M, Saito W, Uchida K (2015). Effectiveness of deep peroneal nerve block for the management of lumbar disease with leg cramps. J. Spine Res.

[b17] Jansen PH, Joosten EM, Vingerhoets HM (1990). Muscle cramp: main theories as to aetiology. Eur. Arch. Psychiatry Neurol. Sci.

[b18] Jansen PH, Lecluse RG, Verbeek AL (1999). Past and current understanding of the pathophysiology of muscle cramps: why treatment of varicose veins does not relieve leg cramps. Eur. Acad. Dermatol. Venereol.

[b19] Kanaan N, Sawaya R (2001). Nocturnal leg cramps. clinically mysterious and painful– but manageable. Geriatrics.

[b20] Khajehdehi P, Mojerlou M, Behzadi S, Rais-Jalali GA (2001). A randomized, double-blind, placebo-controlled trial of supplementary vitamins E, C and their combination for treatment of haemodialysis cramps. Nephrol. Dial. Transplant.

[b21] Lambert EH, Mulder DW (1957). Electromyographic studies in amyotrophic lateral sclerosis. Proc. Staff Meet Mayo Clin.

[b22] Lawrence SJ, Botte MJ (1995). The deep peroneal nerve in the foot and ankle: an anatomic study. Foot Ankle Int.

[b23] Malanga G, Reiter RD, Garay E (2008). Update on tizanidine for muscle spasticity and emerging indications. Expert Opin. Pharmacother.

[b24] Man-Son-Hing M, Wells G, Lau A (1998). Quinine for nocturnal leg cramps: a meta-analysis including unpublished data. J. Gen. Intern. Med.

[b25] Matsumoto M, Watanabe K, Tsuji T, Ishii K, Takaishi H, Nakamura M (2009). Nocturnal leg cramps: a common complaint in patients with lumbar spinal canal stenosis. Spine.

[b26] Miller TM, Layzer RB (2005). Muscle cramps. Muscle Nerve.

[b27] Minetto MA, Holobar A, Botter A, Ravenni R, Farina D (2011). Mechanisms of cramp contractions: peripheral or central generation?. J. Physiol.

[b28] Naylor JR, Young JB (1994). A general population survey of rest cramps. Age Ageing.

[b29] Pitkin RM (1983). Endocrine regulation of calcium homeostasis during pregnancy. Clin. Perinatol.

[b30] Ritchie JM (1975). Mechanism of action of local anaesthetic agents and biotoxins. Br. J. Anaesth.

[b31] Robertson N, Hale W, Mackler L, Poddar S (2005). Clinical inquiries. Does quinine reduce leg cramps for young athletes?. J. Fam. Pract.

[b32] Roffe C, Sills S, Crome P, Jones P (2002). Randomised, cross-over, placebo controlled trial of magnesium citrate in the treatment of chronic persistent leg cramps. Med. Sci. Monit.

[b33] Ross BH, Thomas CK (1995). Human motor unit activity during induced muscle cramp. Brain.

[b34] Sangwan SS, Mittal R, Kundu ZS, Siwach RC, Aggarwal S, Garg RK (2005). Prolapsed intervertebral disc with sciatica: the role of common peroneal nerve block. Trop. Doct.

[b35] Serrao M, Rossi P, Cardinali P, Valente G, Parisi L, Pierelli F (2000). Gabapentin treatment for muscle cramps: an open-label trial. Clin. Neuropharmacol.

[b36] Shaker HK, Mackler L, Huber TE (2005). Clinical inquiries. What is the diagnostic approach to a patient with leg cramps?. J. Fam. Pract.

[b37] Snell RS (2004). Clinical anatomy.

[b38] Sontag SJ, Wanner JN (1988). The cause of leg cramps and knee pains: a hypothesis and effective treatment. Med. Hypothesis.

[b39] Tajiri K, Takahashi K, Ikeda K, Tomita K (1998). Common peroneal nerve block for sciatica. Clin. Orthop. Relat. Res.

[b40] Takayama A, Ito H (2002). Efficacy of deep fibula nerve block in patients suffering from cramps and low back pain. J. Lumbar Spine Disord. (article in Japanese).

[b41] Vinciguerra G, Belcaro G, Cesarone MR, Rohdewald P, Stuard S, Ricci A (2006). Cramps and muscular pain: prevention with pycnogenol in normal subjects, venous patients, athletes, claudicants, and in diabetic microangiopathy. Angiology.

[b42] Warburton A, Royston JP, O’Neill CJ, Nicholson PW, Jee RD, Denham MJ (1987). A quinine a day keeps the leg cramps away?. Br. J. Clin. Pharmacol.

[b43] Yamamoto S (1994). Oral taurine therapy for painful muscle cramp in liver cirrhosis. Am. J. Gastroenterol.

[b44] Young JB, Javid M, George J (1989). Rest cramps in the elderly. J. R. Coll. Physicians Lond.

[b45] Yuda Y, Fujisawa M, Imai K (2005). Peripheral nerve branch block for muscle cramp of the lower legs: anatomy and technique. Pain Clinic (article in Japanese).

[b46] Ziegler D (2008). Painful diabetic neuropathy: treatment and future aspects. Diabetes Metab. Res. Rev.

